# Nutrition and prevention of Alzheimer’s dementia

**DOI:** 10.3389/fnagi.2014.00282

**Published:** 2014-10-20

**Authors:** Arun Swaminathan, Gregory A. Jicha

**Affiliations:** Department of Neurology and Sanders-Brown Center on Aging, College of Medicine, University of KentuckyLexington, KY, USA

**Keywords:** nutrition, Alzheimer, treatment, clinical trial, prevention

## Abstract

A nutritional approach to prevent, slow, or halt the progression of disease is a promising strategy that has been widely investigated. Much epidemiologic data suggests that nutritional intake may influence the development and progression of Alzheimer’s dementia (AD). Modifiable, environmental causes of AD include potential metabolic derangements caused by dietary insufficiency and or excess that may be corrected by nutritional supplementation and or dietary modification. Many nutritional supplements contain a myriad of health promoting constituents (anti-oxidants, vitamins, trace minerals, flavonoids, lipids, …etc.) that may have novel mechanisms of action affecting cellular health and regeneration, the aging process itself, or may specifically disrupt pathogenic pathways in the development of AD. Nutritional modifications have the advantage of being cost effective, easy to implement, socially acceptable and generally safe and devoid of significant adverse events in most cases. Many nutritional interventions have been studied and continue to be evaluated in hopes of finding a successful agent, combination of agents, or dietary modifications that can be used for the prevention and or treatment of AD. The current review focuses on several key nutritional compounds and dietary modifications that have been studied in humans, and further discusses the rationale underlying their potential utility for the prevention and treatment of AD.

## OVERVIEW

Alzheimer’s dementia (AD) is the most commonly recognized cause of dementia in the aging population (Brookmeyer et al., 2007). First described by the German psychiatrist and neuropathologist, Alois Alzheimer, in 1906, the eponymous syndrome is recognized worldwide as a major cause of morbidity and mortality in the aging population contributing to a major burden on healthcare systems (Brookmeyer et al., 2007). Amyloid plaque deposition in the brain, the development of neurofibrillary pathology, neuronal loss and dysfunction of neuroanatomic systems, including those affecting cholinergic transmission, are among the commonly described findings in persons with AD (Jicha and Carr, 2010). In addition to these core pathological features of AD, much evidence has accumulated that increased oxidative stress, defects in mitochondrial dysfunction and cellular energy production, and chronic inflammatory mechanisms contribute to the degenerative cascade in this complex disease (Rao and Balachandran, 2002; Cunnane et al., 2011; Rubio-Perez and Morillas-Ruiz, 2012). A cure for the condition is not known and current treatment approaches include maximizing transmission of acetylcholine and other neurotransmitters while also using other medications and multidisciplinary strategies to treat associated comorbidities focused on improving quality of life and alleviating symptom burden (Jicha and Carr, 2010).

A recent NIH scientific roundtable concluded that there is no definitive evidence for benefit of any preventive strategy for AD, however, this working group also concluded that preventative strategies focused on modifying environmental risk factors deserves further scientific exploration and study (Daviglus et al., 2011). In response to this consensus statement, there was a broad-based push back by many members of the Alzheimer’s research community (particularly from the animal model and epidemiological research folks whose data sets were marginalized and patronized as being of “poor scientific quality”). Researchers in the field of AD were also underrepresented in the consensus group leading to further debate as to the validity of the consensus statement. As such it is clear that the field is far from consensus on the debate of whether viable interventions for the prevention of AD exist. While the search for a cure remains elusive, there is much hope that preventative strategies, such as dietary modification and nutritional supplementation, may reduce the global burden of AD.

**FIGURE 1:**
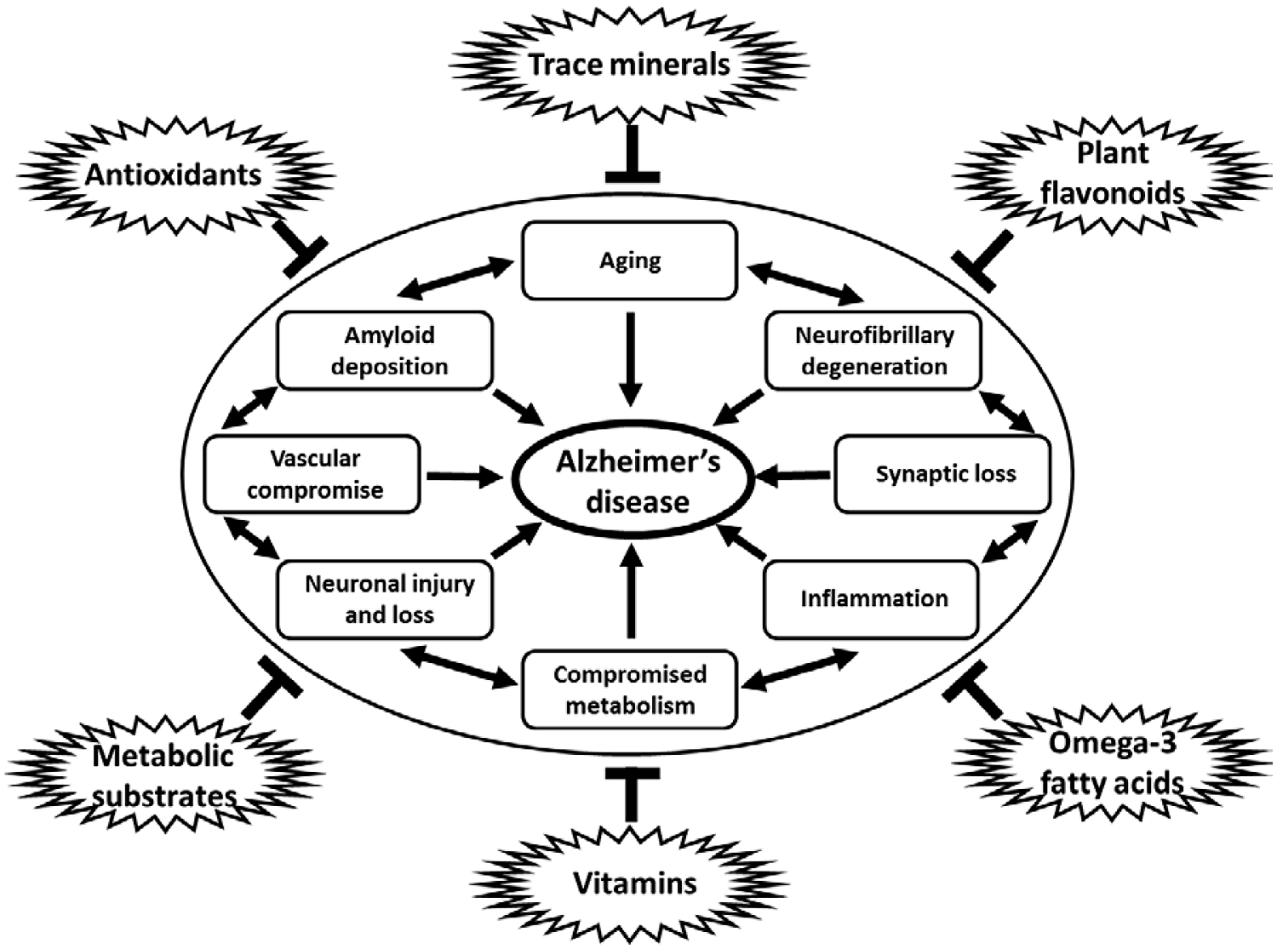
**Diagram of multiple influences of dietary constituents on cellular pathways and process linked to neurodegeneration in AD.** Antioxidants, trace minerals, flavonoids, metabolic substrates and modulators, vitamins, and omega-3 fatty acids, among others, have all been shown to downregulate the many pathological processes linked to the development of AD, including aging, amyloid deposition, neurofibrillary degeneration, synapse loss, inflammation, metabolic compromise, loss of vascular integrity, and neuronal injury and loss. Note: specific dietary factors may have more than one potential mechanism of action on the pathogenic processes contributing to neurodegeneration in AD. Links between pathological processes implicated in the development of AD may not be linear, but rather additive and are shown in a circular fashion without implication for specific linkages or temporal associations between such processes.

A nutritional approach to prevent, slow, or halt the progression of disease is a promising strategy that has been widely investigated. Much epidemiologic data suggests that nutritional intake may influence the development and progression of AD (Gillette-Guyonnet et al., 2013). Modifiable, environmental causes of AD include potential metabolic derangements caused by dietary insufficiency (Knopman et al., 2001; Kamphuis and Scheltens, 2010; Cunnane et al., 2011; Cardoso et al., 2013; Hu et al., 2013; Lopes da Silva et al., 2014). In addition, many nutritional supplements and dietary modifications may directly influence the pathological contributions of increased oxidative stress, defects in mitochondrial dysfunction and cellular energy production, chronic inflammatory mechanisms, and even direct pathways to amyloid accumulation and neurofibrillary degeneration that contribute to the degenerative cascade in AD (**Figure 1**; Rao and Balachandran, 2002; Lau et al., 2007; Weih et al., 2007; Pasinetti and Eberstein, 2008; Kamphuis and Scheltens, 2010; Cunnane et al., 2011; Pocernich et al., 2011; Solfrizzi et al., 2011; Rubio-Perez and Morillas-Ruiz, 2012; Thaipisuttikul and Galvin, 2012; Gillette-Guyonnet et al., 2013; Hu et al., 2013; Shah, 2013). Nutritional modifications have the advantage of being cost effective, easy to implement, socially acceptable and generally safe and devoid of significant adverse events in most cases. Many nutritional interventions have been studied and continue to be evaluated in hopes of finding a successful compound that can be used for the prevention and or treatment of AD (Thaipisuttikul and Galvin, 2012; Hu et al., 2013).

As a discussion of the full complexity of dietary and nutritional interventions for the prevention of AD could fill a several volume book series, for the sake of presenting concise determinate clinical data, the current review focuses on several key nutritional compounds that have been studied in humans, and further discusses the rationale behind their use. Three fundamentally important considerations in interpreting the available human data on nutritional supplementation for the prevention of AD include: (1) Despite our understanding that regulation of metabolic processes are complex and may require multiple nutritional influences to correct aberrant processes leading to AD, traditional experimental design and study to date has largely focused on the use of single agents. Studies of combination therapies and interventions are still in their infancy, but may be extremely important for the identification of truly effective nutritional strategies for the prevention of AD; (2) Considerations of dietary excess and the negative consequences of some dietary constituents (especially those characterizing the current “Western” diet, may interfere with the potentially positive effects of specific nutritional interventions). This has been clearly shown in regards to benefits of omega-3 fatty acids that are neutralized by high dietary intake of omega-6 and saturated fats; (3) Our current understanding of AD includes a prodromal or preclinical phase ranging from 10 to 20 years in which preventative strategies may need to be maintained in order to see their effects. At best, available prevention data is derived from trials with short intervention periods typically extending to 18 months, with few studies extending to a 3 year period (**Table 1**). In addition, there is a relative paucity of “gold-standard,” randomized, double-blind, placebo-controlled, clinical trials investigating the impact of nutritional supplementation in AD. Despite this caveats, researchers, and clinicians remain actively engaged in testing the hypothesis that nutritional approaches may prove benficial for the treatment of AD, while conceding that continued work in this regard is imperative and must remain a high priority for funding authorities across the globe.

**Table 1:** Prospective, randomized, placebo-controlled clinical trials of nutritional interventions in the prevention and treatment of AD

Study intervention	Subjects	Main findings	Important secondary outcomes
Selegiline 10 mg qd; vitamin E 2000 IU qd Sano et al., 1997.	Moderate AD, *n* = 341.	No difference between groups for progression of disease, institutionalization, or death.	Analyses adjusted for entry MMSE scores showed delay to endpoints for selegilene (median 655 days, *p* = 0.012) or vitamin E (median 670 days, *p* < 0.001).
Vitamin E 2000 IU qd; memantine 20 mg qd Dysken et al., 2014.	Mild to moderate AD, *n* = 613.	ADCS-ADL scores decline 3.15 less in vitamin E group than placebo representing a 19% slowing in rate of decline. No effect of memantine.	None.
800 IU vitamin E and 500 mg vitamin C and 900 mg α-lipoic acid; 1200 mg coenzyme Q qd Galasko et al., 2012.	Mild to moderate AD, *n* = 78.	Cerebrospinal fluid F2-isoprostane levels, an oxidative stress biomarker, decreased on average by 19% from baseline to week 16 in the E/C/ALA group but were unchanged in the other groups.	None.
Latrepirdine 5 or 20 mg qd vs. placebo (CONCERT-on donepezil) and CONNECTION Bharadwaj et al., 2013.	Mild to moderate AD, CONCERT, *n* = 1050; CONNECTION, *n* = 598.	No effect on ADAS-Cog outcome measures.	None.
900 mg qd of DHA Yurko-Mauro et al., 2010.	Normal cognition with memory complaints, *n* = 485.	Less PAL six pattern errors with DHA vs. placebo (difference score, -1.63 ± 0.76 [-3.1, -0.14, 95% CI], *p* = 0.03).	Improved immediate and delayed Verbal Recognition Memory scores (*p* < 0.02).
1.7 g DHA and 0.6 g eicosapentaenoic acid vs. placebo Freund-Levi et al., 2006.	Mild to moderate AD, *n* = 204.	No effect on primary outcome measures of MMSE and ADAS-Cog.	Subjects with mild AD, (MMSE > 27 points), showed a significant (*p* < 0.05) reduction in MMSE decline rate.
DHA 2 g qd vs. placebo Quinn et al., 2010.	Mild to moderate AD, *n* = 402.	No effect on primary outcome measures of ADAS-Cog and CDR sum of boxes.	Reduced rate of decline on ADAS-Cog, MMSE, but not CDR sum of boxes for those lacking an ApoE ∊4 allele.
Folic acid 0.8 mg, vitamin B12 0.5 mg and vitamin B6 20 mg vs. placebo Douaud et al., 2013.	Mild cognitive impairment, *n* = 168.	Rate of cerebral atrophy was 3-fold lower in treated vs. placebo group (*p* < 0.001).	The effect was more pronounced for those with homocysteine > 13 mmol/L.
Folate 5 mg, vitamin B6 25 mg, vitamin B12 1 mg qd vs. placebo Aisen et al., 2008.	Mild to moderate AD, *n* = 409	Treatment was effective in reducing homocysteine levels (*p* < 0.001), but had no effect on rate of change in ADAS-cog score.	None.
Subjects received one of two isocaloric conditions (690 calories) in a randomized order: emulsified MCTs, or emulsified long chain triglycerides as a placebo Reger et al., 2004.	AD (*n* = 15) or mild cognitive impairment (*n* = 5).	No effect on primary outcome measures for the composite groups.	In the ∊4-group, there was a significant improvement in ADAS-cog scores following MCT treatment (*F*[1,7] = 6.36, *p* = 0.04). Subjects whose β-OHB levels were higher showed improved paragraph recall with MCT administration (*r* = 0.50, *p* = 0.02).
Oral ketogenic compound, AC-1202 vs. placebo Henderson et al., 2009.	Mild to moderate AD, *n* = 152	ADAS-Cog score on Day 45: 1.9 point difference, *p* = 0.0235 in ITT favoring treatment.	∊4(-) participants (*n* = 55) administered AC-1202 had a significant 4.77 point difference in mean change from Baseline in ADAS-Cog scores at Day 45 (*p* = 0.0005).
Combination supplement with DHA, uridine, choline [Souvenaid®; Kamphuis et al., 2011].	Mild AD, MMSE 20-26, *n* = 225	A significant treatment effect (*F*[1,319] = 4.0, *p* = 0.046) was shown in patients with ``high'' baseline ADAS-cog, but not in patients with ``low'' baseline ADAS-cog (*F*[1,250] = 1.25, *p* = 0.265).	Overall, intake adherence was significantly correlated with ADAS-cog improvement in the active product group (correlation coefficient = -0.260; *p* = 0.019), but not the control group.
Combination supplement with DHA, uridine, choline [Souvenaid®; Shah et al., 2013].	Mild to moderate AD, *n* = 527.	No significant difference in ADAS-cog between study groups [difference = 0.37 points, (standard error) SE = 0.57, *p* = 0.513).	None.
Huperzine A (200 mg BID [*n* = 70] or 400 mg BID [*n* = 70]) vs. placebo Rafii et al., 2011.	Mild to moderate AD, *n* = 210.	No significant difference in change in ADAS-cog scores for Huperazine 200 mg BID (-0.32 ± 15.37 vs. -0.34 ± 5.17, *p* = 0.98).	Huperzine A 400 mg BID treatment effects on ADAS-Cog change demonstrated a non-significant trend 1.92 ± 5.30 point improvement (*p* = 0.07 for the LOCF ANCOVA).
Ginkgo Biloba 120 mg BID vs. placebo DeDea, 2012, Snitz et al., 2009.	Normal adults (*n* = 2587) and MCI (*n* = 482).	Hazard ratio for G. biloba vs. placebo for incidence of dementia was 1.12 (95% confidence interval [CI], 0.94-1.33; *p* = 0.21) and for AD, 1.16 (95% CI, 0.97-1.39; *p* = 0.11).	Rates of change over time in 3MSE, ADAS-Cog, and in neuropsychological domains of memory, attention, visual-spatial construction, language, and executive functions did not differ between groups.

*mg, milligrams; IU, international units; qd, every day; AD, Alzheimer's disease; MMSE, Folstein Mini Mental State Examination score; ADAS-Cog, Alzheimer Disease Assessment Scale-cognitive subtests; POC, proof-of-concept; DHA, docosahexaenoic acid; MCT, medium chain triglycerides; BID, twice daily; MCI, mild cognitive impairment; 3MSE, Modified Mini-Mental State Examination.*

## ANTIOXIDANTS

Increased oxidative stress has been postulated to be a major initiating factor or contributor to the neurodegeneration seen in AD (Filipcik et al., 2006). There is much debate as to the type of antioxidant that may afford the most protection. Some antioxidants preferentially target cytosolic oxidative stress pathways whereas others preferentially serve as mitochondrial cofactors that may reduce intrinsic oxidative stress mechanisms (Filipcik et al., 2006). Antioxidants function in many ways, reducing oxidized membrane lipids, preventing carbonylation of proteins, limiting nucleic acid damage, and influencing stress kinase pathways. Such pleiotropic mechanisms of action inherent in the properties of any given antioxidant preclude precise determination of specific cellular injury or pathway modulation that might be responsible for the beneficial effects of antioxidants in *in vitro* and in animal models of AD. Irrespective of the many potential sites of action and influences on complex cellular pathways influenced by antioxidants, the hypothesis that antioxidant therapy may prevent or slow the development of AD has given rise to many human clinical studies that have provided a much data on the potential risks and benefits of antioxidant therapy in the treatment of AD.

Laboratory research has demonstrated that cytosolic antioxidants, such as vitamin E (α-tocopherol), can prevent AD-like changes in the brains of genetic AD mouse models (Nishida et al., 2006). Other antioxidants such as selegiline, a monoamine oxidaseβ inhibitor, that also inhibits oxidative deamination, have also shown neuroprotective properties in animal models of degenerative disease although definitive human data is lacking in this regard (Tatton, 1993). The Alzheimer’s Disease Cooperative Study Group (ADCS) evaluated these findings in a pivotal clinical trial that examined whether treatment with selegiline or α-tocopherol slowed the progression of disease in patients with moderately severe impairment from AD. Treatment with selegiline (15 mg twice daily) and α-tocopherol (1000 IU twice daily) increased median survival by 215 and 230 days over the placebo, respectively (Sano et al., 1997). These findings led to widespread use of a daily dose of 2000 IU α-tocopherol to slow progression in AD. Widespread use of α-tocopherol supplementation at this dose permeated the field until a meta-analysis suggesting an absolute increase in all-cause mortality of 39/10,000 subjects in the pooled data (*p* = 0.035; absolute risk ratio = 0.0039; number needed to harm = 256) from doses higher than 400 IU per day was published in Miller et al. (2005). The debate on whether vitamin E is protective or deleterious has raged on, with many cautious of the potential adverse effects of such high dose supplementation until 2013 when the ADCS study results were replicated in a large scale “gold-standard” clinical trial, again supporting the use of α-tocopherol at doses of 2,000 IU per day for the treatment of AD (Dysken et al., 2014). Vitamin E should be taken in conjunction with vitamin C as a recharging antioxidant that maximizes the dose of vitamin E. Wheat germ, sunflower, and saﬄower oils, leafy green vegetables, and asparagus are among the best food sources of vitamin E.

In addition to its effects in combination with vitamin E, vitamin C (ascorbic acid) has been widely studied for the prevention and treatment of AD (Bowman, 2012). Vitamin C is an essential vitamin that cannot be produced by humans from glucose or other substrates. Fortunately dietary sources of vitamin C are common and include citrus fruits, berries, and many vegetables that are a common part of most human diets worldwide. Several epidemiologic and cohort studies have investigated the association of vitamin C dietary intake and supplementation with AD and cognitive function in elderly humans. The data from well-characterized cohorts and epidemiologic populations include 33,252 subjects combined and only 3 of these 8 studies have suggested benefit of vitamin C intake (reviewed in Bowman, 2012). Further work examining plasma levels of vitamin C in seven studies (*n* = 1951 combined) showed a significant inverse association of vitamin C with AD and cognitive impairment that was universal across all cohorts studied. Four small studies have looked directly at CSF levels of vitamin C in relation to cognitive performance and AD (*n* = 122 combined), with 3 of the 4 studies showing a positive relationship. These data suggest that dietary intake may be less important than the biological levels attained through dietary intake of vitamin C (reviewed in Bowman, 2012). Unfortunately “gold-standard,” large, multi-center, prospective, randomized, placebo-controlled trials have not been conducted to date. Such studies are needed to determine if prospective supplementation with vitamin C can modify the pathological process responsible for AD. Nonetheless, risks associated with vitamin C supplementation have not been seen in any human studies to date, and the cumulative available data from cross sectional, retrospective cohort, and epidemiological studies suggest that a potential benefit may exist.

Other studies have shown some benefit from mitochondrial cofactors that may function to reduce oxidative stress such as coenzyme Q10 (CoQ10) in reducing amyloid plaque deposition in mice models of AD (Yang et al., 2010). CoQ10 plays a role in the ubiquitin–proteasome complex pathway in the mitochondria that deals with aerobic respiration and cellular breakdown product disposal; a process that is thought to contribute to amyloid plaque formation and accumulation. CoQ10 is easily available and has not associated with any significant adverse events in numerous studies across disparate medical conditions (Hidaka et al., 2008). CSF penetration of CoQ10 is limited however, with maximal availability in the heart, liver, and kidneys (Bhagavan and Chopra, 2006). Nevertheless, it remained a promising agent in the array of antioxidants being used in AD until a recently published head-to-head comparison of cytosolic (α-tocopherol) and mitochondrial (CoQ10) antioxidants was performed in patients with mild to moderate AD (Galasko et al., 2012). The results of this short 4-month study demonstrated benefit of cytosolic antioxidants in reducing isoprostane levels in cerebrospinal fluid, whereas CoQ10 showed no biological benefit in the central nervous system (CNS; Galasko et al., 2012).

Other molecules of recent interest include latreperdine (Dimebon) which was a repurposed antihistamine from Russia that led the field in interest and speculation as a new potential agent for the treatment of AD in the earlier part of this decade (Doody, 2009). The mechanism of action for latreperdine was an enigma until a wealth of data was produced suggesting its mechanism of action was in blocking the mitochondrial membrane pore complex and reducing or protecting mitochondria from the adverse effects of oxidative stress (Bharadwaj et al., 2013). Unfortunately the intrigue and excitement was short-lived as latreperdine soon proved to be ineffective in modifying AD progression in several global Phase-III studies (Bharadwaj et al., 2013). The question as to whether other mitochondrial antioxidants may have utility in the prevention and treatment of AD remains unanswered.

Organic selenium is another antioxidant that is being investigated currently in human clinical studies (Zhang et al., 2010). It is available in several forms, with varied biological actions and efficacy in laboratory paradigms. While inorganic selenium is poorly absorbed, selenomethionine, and yeast-selenium are much more bioavailable after supplementation (Zhang et al., 2010). Animal studies suggest profound effects on brain health and the ability to significantly reduce AD pathology in genetic mouse models (Xiong et al., 2007). These findings have led to the use of antioxidant therapy including vitamins c, e, and selenium in the largest prevention trial of AD ever conducted, the PreADViSe trial, Prevention of AD by Vitamin E and Selenium (Kryscio et al., 2013). This trial remains ongoing and currently no data exists for benefits of selenium supplementation in the prevention or treatment of AD in humans.

Lipoic acid, β-carotene, and other bioflavonoids are just a few of the other antioxidants that have been evaluated for their actions on mitochondrial enzymes like superoxide dismutase and α-ketoglutarate dehydrogenase. Results from clinical trials with these agents have not been sufficient or warranted changes in recommendations for practice. Clearly further work including early stage prevention trials are needed before the utility or lack thereof of antioxidant therapy in the prevention and treatment of AD can be established.

## OMEGA-3 FATTY ACIDS

Fatty acids are found in all the body cells as part of the cell membranes and play a major role in cell membrane stability, fluidity, and synaptic connectivity (Jicha and Markesbery, 2010). Fatty acid oxidation by free radicals results in cell membrane damage and has been postulated to contribute to the pathogenesis of AD, although other roles of unsaturated fatty acids such as lipid raft formation and maintenance of synaptic integrity and function have taken on a primary role in the hypothesis that supplementation with such agents may prevent or slow AD progression (Jicha and Markesbery, 2010). Docosahexaenoic acid (DHA) and eicosapentaenoic acid (EPA) are the major omega-3 fatty acids that have been studied in human clinical trials to date (Jicha and Markesbery, 2010). DHA is the most abundant omega-3 fatty acid in the brain. Polyunsaturated fatty acids (PUFAs) like DHA and EPA allow for lipid raft formation across their unsaturated moieties to absorb the oxidative stress of free radicals and increase cell membrane fluidity necessary for lipid raft creation and formation of effective synaptic contacts (Jicha and Markesbery, 2010). It is currently unclear what the best source of and or combination of specific omega-3 fatty acids might be for the prevention or treatment of AD and so many different sources and formulations have been tested in human clinical trials.

Fish oil, rich in omega-3 fatty acids, when consumed regularly has been found to lower the incidence of AD in epidemiologic studies (Jicha and Markesbery, 2010). Several of these studies have found confounders such as apolipoprotein E (ApoE) allele status and omega-6 intake that have limited the reliability of studies across cohorts (Jicha and Markesbery, 2010). Several studies have demonstrated a lack of association of omega-3 supplementation on a background of a diet high in omega-6 fatty acids, which act similarly to saturated fatty acids decreasing membrane fluidity in opposition to the effects of omega-3 fatty acids (Jicha and Markesbery, 2010). These data suggest that nutritional benefit may be overcome or canceled in the presence of negative dietary contributions. It is intriguing that the dramatic increase in the prevalence of AD over the last century not only parallels the increase in average lifespan, but also an increase from 2 to more than 20 of the ratio of omega-6 to omega-3 PUFAs in the average Western diet (Yehuda et al., 2005). ApoE status may be a further modulator of omega-3 benefit that is discussed further below.

Nonetheless, the epidemiologic and scientific data have led to several large scale clinical trials of omega-3 fatty acids in AD and in normal aging. The MIDAS study found that daily dietary supplementation with 900 mg of DHA produced 7 year age improvement in cognition over just 24 weeks as compared to placebo in elderly patients with cognitive decline (Yurko-Mauro et al., 2010). One large scale trial of fish oil supplementation in Europe showed potential benefit for only those subjects with Folstein Mini-Mental Status scores greater than or equal to 27 (Freund-Levi et al., 2006). Another large scale, multicenter, double-blind, randomized, placebo-controlled trial of high dose DHA supplementation failed to meet primary endpoints for success, but in preplanned secondary analysis a benefit was seen for subjects with mild to moderate AD who were negative for the ApoE ε4 allele (Quinn et al., 2010). The effect of ApoE ε4 responsible for negating the beneficial effects of omega-3 fatty acids is poorly understood, but has been seen in both epidemiologic studies and prospective clinical trials. Carriers of this allele appear to show no benefits of DHA supplementation. This could be due to differences in lipid transport mechanisms or to direct effects on β-amyloid production and clearance. The potential for prevention of AD with omega-3 fatty acid supplementation in non-carriers of the ApoE ε4 allele deserves further study as this population represents nearly 60% of those affected by AD (Jicha and Carr, 2010). Prevention or slowing of the disease process in even this subgroup of sporadic AD cases would have a major impact on the prevalence and health care cost burden associated with AD worldwide. While further work is clearly needed, our current data demonstrates that DHA is well tolerated and has not shown any significant adverse events in these studies and further large scale trials in both normal aging and AD are currently underway.

In addition to potential direct effects on neurodegeneration in AD, the benefits of omega-3 fatty acids for reducing cerebrovascular disease are widely recognized and may provide additional benefit in those suffering from AD, in whom cerebrovascular disease may be the most common comorbidity (Jicha and Carr, 2010). Omega-3 fatty acids reduce circulating cholesterol levels, inhibit systemic inflammation in the circulatory system and vasculature, and inhibit platelet aggregation (Jicha and Markesbery, 2010). The potency of such effects in limiting cognitive decline in AD patients with comorbid cerebrovascular disease may be fundamental for the benefit of omega-3 fatty acids seen in human studies. Further studies aimed at distinguishing direct effects on AD pathogenesis and those associated with the abrogation of cerebrovascular disease are needed to resolve this fundamentally important issue.

## B VITAMINS AND FOLATE

Several studies have shown that elevated serum homocysteine levels and lower vitamin B12 levels are associated with an increased risk of AD (Seshadri et al., 2002; Morris, 2003, 2012; Malaguarnera et al., 2004). Serum folate levels have not shown the same association with AD (Morris, 2002). However, the association of folate with homocysteine metabolism is well known.

Most of the B complex vitamins have been shown to be directly or indirectly associated with neuronal health due to their involvement with neuronal metabolic pathways. Essential functions of the B vitamins include their role in energy metabolism through their action as methyl donors. Deficiency of such vitamins has been directly linked to the development of specific neurological disorders, such as subacute combined degeneration (B12 deficiency), pellagra (vitamin B6 deficiency), Wernicke’s/Korsakoff’s (B1 deficiency). Current clinical recommendations include routine laboratory analysis of such vitamin levels, and repletion if found to be deficient in the setting of cognitive impairment or decline (Knopman et al., 2001). Only limited evidence is available on the effect of supplementation with these vitamins in replete states, although one study found a beneficial effect of B complex vitamin supplementation in slowing the onset or progression of AD in cerebral gray matter although more data analysis is needed to elucidate the exact benefits of these vitamins (Douaud et al., 2013).

Cerefolin NAC is a product containing methylcobalamin, methylfolate and acetylcysteine and is approved for treating vitamin deficiencies associated with memory loss (Thaipisuttikul and Galvin, 2012). Evidence for its use is limited and research is ongoing, despite wide media and consumer appeal. Other clinical trials investigating folic acid and vitamin B supplementation have failed to show convincing results in neurologic improvement despite universal correction of deficiencies and downstream homocysteine levels, and as such the role of high dose B vitamin and folic acid supplementation in the prevention of AD has yet to be established (Aisen et al., 2008; Dangour et al., 2010; Daviglus et al., 2010; Morris, 2012).

## MEDIUM CHAIN TRIGLYCERIDES (AXONA AND COCONUT OIL)

While glucose acts as the primary metabolic substrate in the brain, ketone bodies derived from medium chain triglycerides (MCTs) can serve as an alternative energy source in the brain. Much research has suggested that a localized, brain-specific, insulin resistance develops in AD leading to neuronal dysfunction and death (Craft et al., 2013; de la Monte and Tong, 2014). These data have prompted some to propose ketogenic supplements as a possible strategy for the treatment of AD (Kashiwaya et al., 2013; Zhang et al., 2013; Zilberter et al., 2013).

Patients with memory impairment showed significant improvement in memory after receiving oral supplementation with MCTs that produced higher ketone levels in the blood (Reger et al., 2004). Higher plasma levels of ketones like β-hydroxybutyrate were directly correlated with higher performance in memory scores during this study (Reger et al., 2004). Interestingly enough, the benefits of higher ketone levels were only seen in ApoE4 negative patients during this study, similar to the selective benefits of omega-3 fatty acid supplementation seen in ApoE ε4 non-carriers described above.

Widespread media attention has recently been paid to the notion that coconut oil may be a treatment for AD (DeDea, 2012). This was prompted by the posting of a short video that went “viral” on You-tube showing dramatic improvements in a patient with AD with such dietary modification. The video included commentary from his wife, a pediatrician, who was familiar with the ketone diet used for intractable seizures in this population. The video and case itself has not been published scientifically and it is uncertain if it actually represents true events or is fictitious in nature. In addition, the claim of efficacy for coconut oil use in AD has not been substantiated in any case report in the medical and scientific literature. The postulated mechanism for the effect was the content of caprylic acid, a MCT, that restored brain function as portrayed in this video (DeDea, 2012). Definitive scientific and or clinical evidence for an effect of coconut oil for the prevention or treatment of AD remains to be seen, as no clinical trial data is as of yet available to substantiate or refute these claims.

A nutritional supplement, labeled as a medical food (Axona^®^), containing octanoic acid or caprylic acid has been studied for its potential benefits in AD (Henderson et al., 2009). Supplementation with Axona^®^ in AD patients has been shown to produce improvement in cognition when measured at 45 and 90 days of supplementation (Henderson et al., 2009). Again, the benefits are seen only in ApoE4 ε4 allele negative patients and are short-lived (Henderson et al., 2009). While the benefit remains uncertain, the risks appear minimal, and this strategy is taking root in the armament of treatments many clinicians offer their patients with AD. The most common reasons for discontinuation of Axona^®^ were adverse events like diarrhea, flatulence, and dyspepsia (Henderson et al., 2009). Caution should be exercised before prescribing Axona^®^ or other supplements containing MCTs in patients with diabetes, gastrointestinal inflammation, metabolic syndrome, and renal disturbances as these may amplify the risk of adverse events.

## COMBINATION MEDICAL FOODS (Souvenaid^®^)

Nutritional approaches to influencing the course and progression of AD are among the newer strategies being implemented by scientists. Specially designed medical foods in various combinations are being used to target the underlying process in AD. The rationale behind this approach is that AD results from or causes biochemical alterations in metabolic pathways that are complex and dependent on multiple nutritional compounds and co-factors. Targeting such complex pathways may require combination therapy for maximal success and the use of single agent supplementation may simply be ineffective outside of the rare patient that may have a specific nutritional deficiency. Current innovative approaches include supplying such nutrient combinations in an artificial form to enhance synaptic health and neuronal stability.

Souvenaid^®^ is a medical food designed to contain just such a nutrient combination. Also known as Fortasyn Connect^®^, this medical food is designed to promote synaptic formation and function (Kamphuis et al., 2011; Shah et al., 2013). The product includes precursors (uridine monophosphate; choline; phospholipids; EPA; DHA) and cofactors (vitamins E, C, B12, and B6; folic acid; selenium) necessary for the formation of synapses, integrity of neuronal membranes, resistance to oxidative stress, and optimal metabolic activity in the brain (Kamphuis et al., 2011; Shah et al., 2013).

The existing data have shown mixed results for this approach. One study did not find significant benefits from using Souvenaid^®^ while another showed that patients in the early stage of AD with higher baseline function showed cognitive improvement with higher doses of Souvenaid^®^ (Kamphuis et al., 2011; Shah et al., 2013). Additional trials with refined patient populations and optimized outcome measures may be needed to establish the benefit of this approach.

## DIETARY INFLUENCES ON AD AND DIETARY MODULATION FOR THE PREVENTION OF AD

While the available scientific research and clinical trial data on nutritional interventions in AD have focused on single agents or simple combination therapies, a more holistic approach involves a broader examination of complex dietary influences that may not be easily adapted to scientific study and rigorous clinical examination. Evolutionary Discordance Theory suggests that chronic disease conditions are caused by evolutionary changes in diet (Cordain et al., 2005). This theory has some basis in observational data and on the consideration of the primal hominid diet in the hunter-gatherer Paleolithic era as essential consisting of minimally processed, wild plant and animal foods (Cordain et al., 2005). The rise of many chronic health conditions have paralleled the development of our agrarian culture, now consuming a diet rich in highly processed carbohydrates, refined sugars, alcohols, saturated vegetable oils, dairy products, and fatty domestic meats (Cordain et al., 2005). In light of this theory it is intriguing that an increased risk for AD has been linked to all these evolutionary dietary changes, which include an increase in glycemic index and fatty acid composition of the diet, alterations in macro- and micronutrient composition, increase in sodium intake, and decrease in fiber content (Rao and Balachandran, 2002; Lau et al., 2007; Weih et al., 2007; Pasinetti and Eberstein, 2008; Kamphuis and Scheltens, 2010; Cunnane et al., 2011; Pocernich et al., 2011; Solfrizzi et al., 2011; Rubio-Perez and Morillas-Ruiz, 2012; Thaipisuttikul and Galvin, 2012; Gillette-Guyonnet et al., 2013; Hu et al., 2013; Shah, 2013). Such dietary changes have been linked to the rise in prevalence of metabolic syndrome that has been further associated with the rise in AD prevalence in western society.

Metabolic syndrome is defined by the presence of at least three of the following: abdominal or central obesity (waist circumference >102 cm for men and >88 cm for women); elevated plasma triglycerides (TGs; ≥150 mg/dl); low high-density lipoprotein (HDL) cholesterol (<40 mg/dl for men and <50 mg/dl for women); high BP (≥130/≥85 mm Hg) or normotensive on hypertensive treatment; high fasting plasma glucose (≥110 mg/dl) or euglycemic on antidiabetic treatment (Grundy et al., 2005). This combination of central obesity, hyperlipidemia, hypertension, and insulin resistance or type II diabetes has been strongly linked with increased risk for development of AD (Frisardi et al., 2010). The metabolic syndrome has been clearly associated with increased risk for cerebrovascular disease which is a substantial comorbidity and confounding contributor to the development of AD in community based cohorts, but also may directly increase systemic and CNS inflammation processes linked to AD through induction of adipokines that modulate these processes (Panza et al., 2010; Solfrizzi et al., 2010). While the components of metabolic syndrome are highly dependent on diet, contributions of other lifestyle factors leading to the metabolic syndrome, such as reduced exercise, should not be minimized (Grundy et al., 2005). While several studies examining pharmacologic manipulation of hypertension and hyperlipidemia have demonstrated cognitive benefit in the aging population, no interventional studies focused on nutritional correction of the complete metabolic syndrome in the prevention of AD exist in the literature today (Tzourio et al., 2003; Masse et al., 2005; Rockwood, 2006). Despite such positive results, large scale, multi-center, randomized, placebo-controlled human clinical trials of statins to reduce cognitive decline associated with AD have failed to date, leaving many to question the potential impact of modulating a single or isolated component of the metabolic syndrome in light of the complex, multifaceted, pathways involved in the development of AD (Sano et al., 2011).

Caloric restriction may counter the negative consequences of metabolic syndrome, has been investigated widely, and has shown cross species benefits in slowing aging process and extending expected lifespan (Gillette-Guyonnet and Vellas, 2008). The brain benefits of caloric restriction may be attributable to induction of neurogenesis and enhancement of synaptic plasticity, potentially protecting the brain from age-related senescence and allowing increased recovery and compensation following neuronal injury or in the face of degenerative processes such as AD (Gillette-Guyonnet and Vellas, 2008). While animal data is robust, little human data has been acquired to date on the role of caloric restriction in preventing AD.

People living in the Mediterranean region, especially Naples and the surrounding parts of Italy, have been found to have the highest life expectancies in the world and have also been found to have lower incidence of AD. This observation has convinced many authorities to attribute this to their dietary habits. The Mediterranean diet is extremely well known and popularly recommended as a preventive measure for AD and cognitive impairment (Feart et al., 2010; Sofi et al., 2010). Although there is no fixed recipe for this diet, generally speaking, a diet with generous servings of fruits, vegetables, whole grains, beans, nuts, seeds with low to moderate amounts of fish, poultry, dairy products, and low amounts of red meat is seen in most variations of this diet. Such diets have been shown to modulate inflammatory, oxidative stress-induced, regenerative, and cellular health processes that may play critical roles in modulating risk for AD. Olive oil forms an important component of this diet and is rich in monounsaturated fatty acids (MUFAs) and oleocanthal, the latter being shown to have inhibitory properties on fibrillization of tau protein, thus conferring it with potentially protective properties in AD (Monti et al., 2011). Epidemiologic studies have shown that higher adherence to a Mediterranean-style diet results in reduced mortality in patients with AD in a dose responsive manner with greater compliance and dosage being associated with greater benefits (Scarmeas et al., 2007). Research has also shown that greater consumption of Mediterranean diet with higher compliance was associated with a lower risk of AD (primary prevention) and slower progression of symptoms (secondary prevention) of mild cognitive decline into AD (Scarmeas et al., 2006; Solfrizzi et al., 2011).

The benefits of the Mediterranean diet have been attributed to the presence of MUFA and PUFA in addition to the right mix of micro and macronutrients that promotes neuronal health and minimizes AD risk. “Gold-standard” clinical trial is not yet available for such dietary interventions given the complexity of practical implementation. Additional considerations not answered by the epidemiologic data include possible effects of genetic background (ApoE status as seen for other nutritional approaches), duration of dietary modification needed before benefit may be evident, and confounding lifestyle factors that may influence response to such dietary approaches or influence the outcome disease state and processes. Despite these caveats, the adage “You are what you eat” may hold true and the health benefits of a Mediterranean-style diet are well accepted across multiple disciplines of medicine with no adverse potential or limitations for the general population. Only limited studies of other diets have been published to date with no clear evidence emerging.

Unfortunately, as clinical interventions of total dietary intake and composition are limited by the complexity of the human diet, the best available data for dietary influences in the prevention of AD come from retrospective cohort and epidemiologic studies rather than from prospective clinical trials. This is in large due to the many confounds inherent in total dietary regulation required to establish causality and the potential benefit of prospective dietary modification. With these caveats in mind, it is still worthwhile to consider the emerging evidence that modification of dietary patterns, including caloric restriction, and avoiding the current “Western” diet, may prove to be effective strategies for the prevention of AD.

## OTHER NUTRITIONAL SUPPLEMENTS EVALUATED IN HUMAN CLINICAL TRIALS

Apart from the nutritional substances mentioned above, a myriad of other natural supplements have potential to modify the development and progression of AD, although definitive evidence for efficacy is still lacking. Many of these agents are commonly used in traditional medicine culture in India, China, and other areas of Eastern Asia and have generated a great deal of interest in their potential therapeutic benefit for AD in Western society.

Huperzine A is a traditional Chinese herb, with pharmacological actions as an acetylcholinesterase inhibitor (AChEI) among other properties that has been evaluated in several small clinical trials that have suggested potential benefit in AD patients (Yang et al., 2013). In the laboratory, huperzine A has been shown to reduce amyloid plaque formation and abrogate cell death by altering neuronal iron content in animals models of AD (Huang et al., 2014). A recent multicenter clinical trial of Huperzine A failed to meet primary endpoints for success suggesting its potential benefits may be limited in human disease, however, the study suffered from several limitations, including the concomitant use of pharmacological AChEI that have diluted the effects of the herb that would otherwise have been seen in drug-naïve populations (Rafii et al., 2011).

Gingko biloba, another Chinese herb, has also been studied for potential benefit in AD. It contains ginkgolide B, a platelet activating factor (PAF) antagonist, and has been used in stroke trials due to this property. A large scale primary and secondary prevention study analyzed the efficacy of gingko biloba in preventing the development of or slowing progression of mild cognitive impairment into AD and found no benefits (DeKosky et al., 2008; Snitz et al., 2009). Despite this failure, it continues to be a popular treatment for a variety of medical conditions and may yet hold promise for the treatment of vascular cognitive impairment and dementia related to other non-AD causes.

Resveratrol is a polycyclic aromatic compound found in skins of grapes, raspberries, mulberries in varying concentrations that has been found to have powerful antioxidant properties (natural plant polyphenol; Pervaiz and Holme, 2009). Laboratory studies have shown multiple mechanisms of action including function as a powerful antioxidant, decreased amyloid plaque deposition in animal models of AD, and inhibition of intracellular inflammatory pathways that predispose to cellular breakdown and cell death (Anekonda, 2006; Pervaiz and Holme, 2009). Perhaps the most interesting data of all links resveratrol to sirtuin pathways that are key mediators of cellular aging processes, suggesting it may promote longevity (Anekonda and Reddy, 2006). Animal studies have demonstrated that resveratrol improves the health and survival of mice fed a high calorie diet (Baur et al., 2006). Thus, resveratrol may not only influence AD pathways, but may also enhance metabolic activity, thereby reducing risks for obesity and cerebrovascular disease that can contribute to the development of dementia, in addition to reductions in cellular aging processes (Baur et al., 2006). As age remains the major risk factor for AD, resveratrol is being tested currently in a multicenter clinical trial in mild to moderate AD patients in addition to a widespread range of other age-related diseases^1^. Available safety data suggests that supplementation with resveratrol is relatively benign although no current recommendations as to its usefulness van be made without more human data including the results of the ongoing trial.

Turmeric is a spice used commonly in eastern cuisines and has been associated with antioxidant properties that have spiked interest in its role on treating or preventing cancer, aging and AD. Curcumin, the active ingredient of turmeric, has been shown to have antioxidant scavenging properties and increase glial fibrillary acid protein expression in hippocampi of mice and increase spatial memory (Wang et al., 2013; Fang et al., 2014). Curcumin exhibits powerful anti-inflammatory effects through its downregulation of proinflammatory transcription factors including nuclear factor-kappa B, signal transducer and activators of transcription-3, and Wnt/beta-catenin (Aggarwal, 2010). Curcumin has additionally been shown to directly reduce amyloid plaque formation and associated inflammation in the brains of AD Mouse models (Wang et al., 2013). Two studies have published or presented the data from these trials to date, both showing excellent safety profiles of curcumin, but neither showing benefit in regards to clinical efficacy or influences on traditional AD biomarkers (Baum et al., 2008; Ringman et al., 2008). Several other human trials are currently underway^2^, however, definitive data is not yet available on the influence of this nutritional agent in the prevention or treatment of AD in humans.

## SUMMARY

A nutritional approach to preventing AD appears to be an innovative and safe approach that may be extremely cost effective, allow ease of administration, and importantly, serve as a socially acceptable intervention or adjunctive approach in the prevention and treatment of AD. Despite years of scientific, medical, and clinical advances in this area, much remains to be discovered and proven in terms of specific nutritional interventions for the prevention of AD. Promising agents such as vitamins, energy substrates, flavonoids, lipids, and modified diets functioning as anti-oxidants, metabolic-enhancers, immune-modulators, and direct disease-modifying agents await further investigation. To date no definitive evidence for disease modification outside of animal and *in vitro* experiments exist, and yet human clinical data is beginning to suggest that such interventions deserve further study (**Table 1**).

Yet it is possible, despite the wealth of retrospective and prospective human data available that nutritional interventions for the prevention of AD may be effective, it is equally possible that they may serve to only supplement direct disease-modifying treatment, and in themselves are ineffective at modulating the AD disease state. Human evolutionary dietary data does not take into account the extended lifespan see in modern day humans, and so evidence supporting evolutionary reversion of diet may be misleading. Evidence from studies of omega-3 fatty acids like DHA and energy substrates like Axona^®^ suggest that disease modulation of nutritional intervention may be genetically modulated (Reger et al., 2004; Quinn et al., 2010). It is possible that dietary modulation and nutritional supplementation serves only to bolster normal health mechanisms that are a natural deterrent of chronic health conditions such as AD without really possessing any discrete disease specificity. Future investigations in the area of nutritional and dietary prevention of AD will have to consider these potential confounds and overcome them if nutritional and dietary supplementation and modification are ever to become part of the clinical care paradigm for the prevention and treatment of AD.

While the field of interventional nutrition in AD is expanding at an exciting pace and the latest developments in this field are being closely followed by researchers, clinicians, the public, and the lay media, definitive clinical trials are lacking secondary to funding limitations and opportunities, adherence to traditional empiric clinical methodologies that seek to investigate single mechanisms of disease pathogenesis and intervention, and the development of strategies to overcome basic issues of control populations and incorporation of often complex interventional strategies for the multi-faceted approaches that may be required to move the investigation of nutritional interventions for the prevention of AD forward.

Given the complexity of the field in regards to nutritional interventions for the prevention of AD and the considerations and limitations an available to date, it is important to draft recommendations for advancing the field in this regard. Recommendations stemming from this review include: (1) Expanding research funding opportunities beyond those available from the National Center for Complementary and Alternative Medicine (NCCAM), to include funding from other NIH centers and to encourage state-of-the-art and “gold-standard” research from industry and private organizations; (2) Design of trial methodology and data analysis techniques to account for complexities in dietary patterns that may influence investigations of single nutriceutical agents or simple combinations of such agents, that may be amenable to the desired goal of rigorous prospective scientific investigation of comprehensive dietary alterations in the prevention of AD; (3) Incorporation of longer trial periods reflecting our increased understanding of the extensive (10–20 year) prodromal period of AD where prevention may be most effective; and (4) Broad scientific discovery from biological samples derived from prospective clinical interventions focused on investigating the basic mechanisms underlying the potential beneficial effects of nutritional interventional strategies including metabolomic, antioxidant, immunologic, neuroprotective and cellular regenerative discoveries. The development of a fundamental framework incorporating such features is necessary for advancing the field of nutritional interventional strategies for the prevention and treatment of AD.

## Conflict of Interest Statement

The authors declare that the research was conducted in the absence of any commercial or financial relationships that could be construed as a potential conflict of interest.

## Footnotes

1 http://www.clinicaltrials.gov/ct2/show/NCT01504854

2 http://www.clinicaltrials.gov/ct2/results?term=curcumin+and+alzheimer
